# High-quality draft genome sequence of a new phytase-producing microorganism *Pantoea sp*. 3.5.1

**DOI:** 10.1186/s40793-015-0093-y

**Published:** 2015-11-11

**Authors:** Aliya D. Suleimanova, Anna A. Toymentseva, Eugenia A. Boulygina, Sergey V. Kazakov, Ayslu M. Mardanova, Nelly P. Balaban, Margarita R. Sharipova

**Affiliations:** Laboratory of Biosynthesis and Bioengineering of the Enzymes, Institute of Fundamental Medicine and Biology, Kazan (Volga region) Federal University, Kazan, Russia; Interdisciplinary Center for Proteomics Research, Kazan (Volga region) Federal University, Kazan, Russia; Omics Technologies Laboratory, Kazan (Volga region) Federal University, Kazan, Russia; Computer Technologies Laboratory, Information Technologies, Mechanics & Optics University, Saint Petersburg, Russia

**Keywords:** Strain 3.5.1, *Pantoea*, Genome, 454, Ion Torrent, Phytase

## Abstract

Strain 3.5.1 was isolated from soils of the Republic of Tatarstan, Russia, on the basis of presence of a high phytate-degrading activity. Strains with such activities attract special interest because of its potential use as feed additives and natural manures. Strain 3.5.1 harbors a 99 % 16S rRNA nucleotide sequence similarity to different *Pantoea* species (*P. vagans*, *P. ananatis*, *P. agglomerans*, *P. anthophila* and *Pantoea sp*.) and exhibits unique biochemical properties that do not allow strain identification up to species. Moreover, the strain 3.5.1 shows a low ANI and MALDI-TOF Mass Spectrometry scores. Thus, it is likely that the strain 3.5.1 represents a new *Pantoea* species. Here, we present the genome sequence of *Pantoea sp*. strain 3.5.1. The 4,964,649 bp draft genome consists of 23 contigs with 4,556 protein-coding and 143 RNA genes. Genome sequencing and annotation revealed two phytase genes and putative regulatory genes controlling its activity.

**Electronic supplementary material:**

The online version of this article (doi:10.1186/s40793-015-0093-y) contains supplementary material, which is available to authorized users.

## Introduction

Up to 90 % of natural phosphorus in the World is present in the form of phytic acid or phytate and is often accumulated in livestock feces. This form of organic phosphorus cannot be utilized by monogastric farm animals and ends up polluting soils and contributes to the eutrophication of water environments [1, 2]. Moreover, phytate reduces the nutritional value of feeds because it chelates essential minerals such as calcium, iron, zinc, magnesium, manganese, copper and molybdenum [3]. Chemical (acid hydrolysis ion and exchange) or physical (autoclaving) methods to hydrolyze phytate are costly and reduce the nutrient value of feeds. Therefore, the search for of alternative methods of phytate hydrolysis is an important task. In light of this, identification and isolation of bacteria capable of enzymatic phytate hydrolysis is a promising approach that would simultaneously reduce environmental burden caused by current agricultural practices.

Phytases are specific group of phosphatases capable of phytate (myo-inositol 1,2,3,4,5,6-hexakisphosphate) hydrolysis with the formation of less phosphorylated inositol derivatives [4, 5]. There are a few reports on phytase-producing microbes from Russia; they include fungi [6, 7] and bacteria [5, 8, 9]. Here, we characterize a phytase-producing strain 3.5.1, present its classification and describe a set of its features along with the annotated genome sequence that provides important insights into several candidate genes involved in phytate hydrolysis. Strain 3.5.1 was isolated from a forest soil sample on a selective medium containing calcium phytate as the only source of phosphorus.

## Organism Information

### Classification and features

The genus *Pantoea*, within the *Enterobacteriaceae* family, consists of several species (*P. agglomerans*, *P. ananatis*, *P. dispersa*, *P. vagans* and others) that generally inhabit numerous ecological niches, including plants, water, soil, humans and animals. Classification of these species had a long history before they were separated in the new *Pantoea* genus [10]. *P. agglomerans* (formerly *Enterobacter agglomerans*) and *P. dispersa* were proposed as the first *Pantoea* species based on their DNA–DNA hybridization relatedness. Mergaert et al. proposed the name *P. ananatis* for *Erwinia ananas* [11]. Brady et al. isolated *P. agglomerans*-like strains and separated them into four novel species (*P. vagans*, *P. eucalypti*, *P. deleyi* and *P. anthophila*) based on MultiLocus Sequence Analysis and amplified fragment length polymorphism analysis [12]. Identification of *Pantoea* species through their nutritional characteristics or biochemical approaches has proven to be difficult. Currently, several strategies based on the use of genomic approaches have been reported to define *Pantoea* species [13–16]. One of challenging approaches to construct the phylogenetic relationships among different bacterial isolates is a whole genome sequencing [17]. To date the NCBI database contains information about nine of 23 validly published *Pantoea* species genome assemblies.

**Fig. 1:**
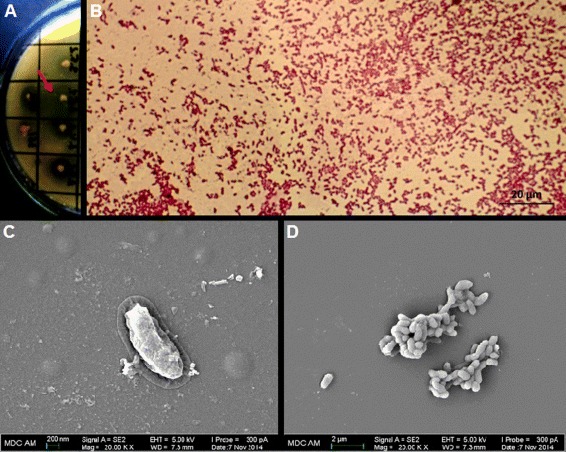
General characteristics of the strain 3.5.1. **a** Screening for phytate-hydrolyzing activity on PSM solid medium. Strain 3.5.1 is indicated by red arrow. **b** Phase contrast micrograph of the strain 3.5.1. **c** and **d** Scanning electron micrographs of the strain 3.5.1 (Carl Zeiss, Merlin)

Strain 3.5.1 was isolated from the forest soil near Agerze village, Aznakaevo district, Republic of Tatarstan, Russia [18, 19]. The isolate was characterized as Gram-negative, motile and rod-shaped bacterium 0.5 μm to 1.5 μm length (Fig. 1 and Table 1). Colonies were round, smooth and shiny after incubation at 37 °C for 24 h. Longer incubation (2–3 days) of the isolate resulted in production of yellow pigment. The strain 3.5.1 displayed phytate-hydrolyzing activity on PSM solid medium (2 % Glucose, 0.4 % Sodium phytate, 0.2 % CaCl_2_, 0.5 % NH_4_NO_3_, 0.05 % KCl, 0.05 % MgSO_4_ × 7H_2_O, 0.001 % FeSO_4_ × 7H_2_O, 0.001 % MnSO_4_ × H_2_O, 3 % Agar, pH 7.0), *i.e.* it was able to form halo zone around the colonies (clear zone reflecting solubilization of Ca-phytate in the agar medium) (Fig. 1A) [20, 21]. Thus, strain 3.5.1 has the unique characteristics of degradation of phytate and can potentially be used for the industrial production of microbial phytase; the enzyme could possibly be applied as phosphorus-mobilizing agent in soil or as a feed supplement for livestock production.

**Table 1:** Classification and general features of *Pantoea sp*. 3.5.1. in accordance with the MIGS recommendations [42] as published by the Genome Standards Consortium [43]

MIGS ID	Property	Term	Evidence code^a^
	Classification	Domain *Bacteria*	TAS [44]
		Phylum *Proteobacteria*	TAS [45]
		Class *Gammaproteobacteria*	TAS [45, 46]
		Order *“Enterobacteriales”*	TAS [47–49]
		Family *Enterobacteriaceae*	TAS [49]
		Genus *Pantoea*	TAS [10, 50]
		Species *Pantoea sp.*	TAS [51, 52]
		Type strain: 3.5.1	IDA
	Gram stain	Negative	IDA
	Cell shape	Rod-shaped	IDA
	Motility	Motile	IDA
	Sporulation	Non-spore forming	IDA
	Temperature range	Mesophilic	IDA
	Optimum temperature	+37 °C	IDA
	pH range; Optimum	3.5–7; 5	IDA
	Carbon source	D-glucose, lactose, maltose, mannite	IDA
	Energy source	Chemoorganotroph	NAS
MIGS-6	Habitat	Soil	IDA
MIGS-6.3	Salinity	Not tested	
MIGS-22	Oxygen requirement	Facultative aerobic	IDA
MIGS-15	Biotic relationship	Free living	IDA
MIGS-14	Pathogenicity	Opportunistic pathogen	NAS
MIGS-4	Geographic location	Agerze village, Aznakaevo district, Republic of Tatarstan, Russia	IDA
MIGS-5	Sample collection	September 2010	IDA
MIGS-4.1	Latitude	54°83´	IDA
MIGS-4.2	Longitude	53°00´	IDA
MIGS-4.3	Depth	25 cm	IDA
MIGS-4.4	Altitude	233 m	IDA

^a^Evidence codes - IDA: Inferred from Direct Assay; TAS: Traceable Author Statement (*i.e.*, a direct report exists in the literature); NAS: Non-traceable Author Statement (*i.e.*, not directly observed for the living, isolated sample, but based on a generally accepted property for the species, or anecdotal evidence). These evidence codes are from the Gene Ontology project [53]

Strain 3.5.1 was shown to be able to utilize the following carbon substrates: glucose, lactose, maltose and mannitol without gas formation, but unable to oxidize urea (tested on Kligler Iron Agar, Olkenitski's medium and Hiss media) [22, 23]. By API-20E test (bioMerieux, Inc.) it was shown that the strain 3.5.1 cannot utilize ornithine. The strain is resistant to tetracycline, chloramphenicol and erythromycin but susceptible to beta-lactam antibiotics like ampicillin and penicillin. These morphological and biochemical properties are consistent with the notion that this isolate likely belongs to the family *Enterobacteriaceae*.

**Fig. 2:**
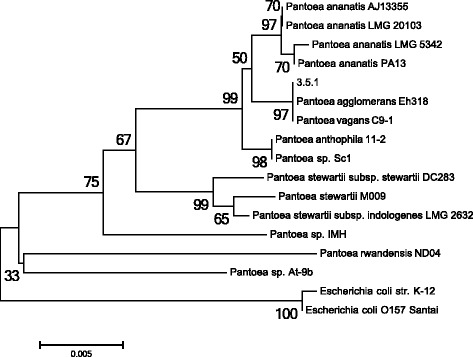
Maximum likelihood phylogenetic tree highlighting the position of the strain 3.5.1 relative to other species within the genus *Pantoea* based on 16S rRNA gene sequences. Bootstrap consensus tree were inferred from 1,000 replicates. Two *E. coli* strains were used as outgroup. The scale bar, 0,005 substitutions per nucleotide position. The phylogenetic tree was obtained by MEGA 6 software [44]. The corresponding GenBank accession numbers for 16S rRNA sequences are: NR_074740 (*P. ananatis* AJ13355), FJ611814 (*P. ananatis* LMG 20103), FJ611845 (*P. ananatis* LMG 5342), NC_017554 (*P. ananatis* PA13), NZ_JMRT02000019 (the strain 3.5.1), NZ_KK403348 (*P. agglomerans* Eh318), NR_102966 (*P. vagans* C9-1), JXXL01000005 (*P. anthophila* 11–2), FJ611810 (*Pantoea sp*. Sc1), AJ311838 (*P. stewartii* subsp. *stewartii* DC283), KJ830125 (*P. stewartii* M009), NR_119256 (*P. stewartii* subsp. *indologenes* LMG 2632), JX861128 (*Pantoea sp*. IMH), NZ_CP009454 (*P. rwandensis* ND04), NC_014837 (*Pantoea sp*. At-9b), NR_102804 (*E. coli* K-12 substr. MG1655), NZ_CP007592.1 (*E. coli* O157:H16 strain *Santai*)

The taxonomic position of the strain 3.5.1 was first evaluated by the comparison of 16S rRNA gene sequences with related sequences using blastn (nr/nt GenBank Database). The sequence showed 99 % identity to multiple 16S sequences from *Pantoea* species (*Pantoea**spp*., *P. ananatis*, *P. vagans*, *P. agglomerans*, *P. conspicua* and others). More detailed phylogenetic analysis of the strain 3.5.1 was performed using MEGA 6.0 software [24] with 16S rRNA gene sequences of 21 *Pantoea* species and 2 *Escherichia coli* strains as an outgroup (a complete/scaffold level genome sequences for all these species are available in NCBI database). However, our alignment allowed comparison of only variable regions V3 and V4 of 16S rRNA gene for these set of species, because not all completed sequences of these genes are available. Therefore, we eliminated several species from phylogenetic comparison to generate a tree based on the extended variable regions of 16S rRNA gene [25, 26]. Finally, 14 *Pantoea* species and 2 *Escherichia coli* strains were aligned, the incomplete sites on both 5′- and 3′-ends of the 16S rRNA gene sequences were excluded from the alignment. The remaining alignment sites (1208 bp), which included V1–V8 regions of 16S rRNA sequences, were selected for the subsequent analysis. Phylogenetic tree was generated using the Maximum likelihood (ML) algorithm with 1,000 bootstrap iterations (Fig. 2). As expected, two strains of *E. coli* (K-12 substr. MG1655 and O157:H16 Santai) could be clearly distinguished phylogenetically from species that belong to *Pantoea* genus. *P. ananatis* and *P. stewartii* belong to two different clades of the tree with high bootstrap support. However, certain clades, such as *P. agglomerans*, *P. vagans* and *Pantoea**sp*., do not form clearly separate groups. Interestingly, despite the fact that the strain 3.5.1 forms a distinct node with *P. agglomerans* Eh318 and *P. vagans* C9-1, *P. vagans* species do not show motility at 37 C° and both with *P. agglomerans* strains are not able to hydrolyze lactose as a carbon source, unlike the strain 3.5.1 [12]. We also carried out the matrix-assisted laser-desorption/ionization time-of-flight MS protein analysis for the strain 3.5.1 using a Microflex spectrometer (Bruker Daltonics, Leipzig, Germany). Measurements were made as previously described [27]. Spectra of the strain 3.5.1 were imported into the MALDI BioTyper software (version 2.0; Bruker) and analyzed by standard pattern matching (with default parameter settings). The commercially available Bruker database contains 26 protein profiles of *Pantoea* species and 14 protein profiles of *E. coli* species. All these profiles were used as reference data to compare the strain 3.5.1 spectra. Strain 3.5.1 showed log (score) values between 1.6 and 2.0 which allowed defining only its genus identification. The 3.5.1 spectra obtained are shown as a dendrogram in Additional file 1. We then calculated average nucleotide identity values between the genome sequences of the strain 3.5.1 and 21 *Pantoea* species using the JSpecies software [28] based on the BLASTn method (ANIb) and the MUMmer algorithm (ANIm). Analysis of the 3.5.1 genome data yielded low ANI values (ANIb 74.88-85.11 % and ANIm 83.72-86.86 %) indicating that the strain 3.5.1 does not belong to previously characterized species (Additional file 2), since the lowest threshold for ANI clustering is 97 %. Since the strain 3.5.1 differs from known *Pantoea* species both biochemically and phylogenetically we designate our isolate as *Pantoea**sp*. 3.5.1.

## Genome sequencing information

### Genome project history

The genome of *Pantoea**sp*. strain 3.5.1 was selected for whole genome sequencing because of its ability to produce phytase. Comparison of the strain 3.5.1 genome with other *Pantoea* species may provide insights into the molecular basis of phytase activity and metabolic features of this strain. The high-quality draft genome sequence was completed on March 27, 2015 and was deposited to GenBank as the Whole Genome Shotgun project under the accession number JMRT00000000 (current version JMRT00000000.2) and to the Genome OnLine Database with ID Gp0114842 [29]. A summary of the project information is shown in Table 2.

**Table 2:** Project information

MIGS ID	Property	Term
MIGS 31	Finishing quality	High quality draft
MIGS-28	Libraries used	Two single-end libraries of 200 bp and 600 bp
MIGS 29	Sequencing platforms	Ion Torrent PGM and 454 GS Junior
MIGS 31.2	Fold coverage	32×
MIGS 30	Assemblers	SPAdes 3.5.0
MIGS 32	Gene calling method	GeneMark, RAST
	Locus Tag	EP46
	Genbank ID	JMRT00000000.2
	GenBank Date of Release	April 16, 2015
	GOLD ID	Gp0114842
	BIOPROJECT	PRJNA246264
MIGS 13	Source Material Identifier	3.5.1
	Project relevance	Phytase producer bacterium, Agricultural

### Growth conditions and genomic DNA preparation

The *Pantoea**sp*. strain 3.5.1 is deposited to the Russian National Collection of Industrial Microorganisms (VKPM) under the accession number В-11689.

For genomic DNA isolation bacterial culture was grown overnight in 25 mL LB medium at 37 °C with vigorous shaking. DNA was isolated using a Genomic DNA Purification Kit (Fermentas). DNA purity was tested by gel electrophoresis (1 % agarose gel) and DNA concentration was estimated by the Qubit 2.0 Fluorometer using the Qubit dsDNA (High Sensitivity) Assay Kit (Life Technologies).

### Genome sequencing and assembly

The genomic DNA of *Pantoea**sp*. 3.5.1 strain was sequenced with 32-fold overall genome coverage by a whole genome shotgun strategy. Two single-end libraries were used: a 200 bp-library for Ion Torrent PGM sequencing (performed in the Research Institute of Physical Chemical Medicine, Moscow, Russia) and 600 bp library for 454 GS Junior sequencing (performed in the Interdisciplinary Center for Proteomics Research, Kazan, Russia). Sequencing of the 200 bp library generated 349,046 reads, while sequencing of the 600 bp library generated 152,266 reads. Both read sets were assembled *de novo* using the SPAdes 3.5.0 assembler [30]. This strategy resulted in 23 contigs (>500 bp) with a calculated genome size of 4,964,649 bp and G + C content of 55,77 mol %. The N50 size of the resulted contigs was 562,444 bp.

### Genome annotation

Genes of *Pantoea**sp*. 3.5.1 strain were identified using the Prokaryotic Genomes Automatic Annotation Pipeline. The predicted CDSs were translated and analyzed against the NCBI non-redundant database, iPfam, TIGRfam, InterPro, KEGG, COG and IMG databases [31–36]. The genome sequence was also uploaded into the RAST system [37] to check the annotated sequences.

## Genome properties

The draft assembly of the genome consists of 23 contigs with the fragment size lager than 500 bp, N50 is 562,444 bp. Of the 4,699 genes predicted, 4,556 were protein-coding genes and 143 were RNA genes. Putative functions were assigned to the majority of the protein-coding genes (96.96 %), while the remaining ORFs (open reading frames) were annotated as hypothetical proteins. The distribution of genes into COGs functional categories is presented in Tables 3 and 4.

**Table 3:** Genome statistics

Attribute	Value	% of Total
Genome size (bp)	4,964,649	100.00
DNA coding (bp)	4,306,583	86.74
DNA G + C (bp)	2,768,589	55.77
DNA scaffolds	23	100.00
Total genes	4,699	100.00
Protein coding genes	4,556	96.96
RNA genes	143	3.04
Pseudo genes	135	2.87
Genes in internal clusters	NA	
Genes with function prediction	3,921	83.44
Genes assigned to COGs	3,507	74.63
Genes with Pfam domains	4,059	86.38
Genes with signal peptides	425	9.04
Genes with transmembrane helices	1,075	22.88
CRISPR repeats	1	-

**Table 4:** Number of genes associated with general COG functional categories

Code	Value	%age	Description
J	250	6.28	Translation, ribosomal structure and biogenesis
A	1	0.03	RNA processing and modification
K	366	9.19	Transcription
L	127	3.19	Replication, recombination and repair
B	-	-	Chromatin structure and dynamics
D	45	1.13	Cell cycle control, Cell division, chromosome partitioning
V	85	2.13	Defense mechanisms
T	186	4.67	Signal transduction mechanisms
M	250	6.28	Cell wall/membrane biogenesis
N	106	2.66	Cell motility
U	48	1.21	Intracellular trafficking and secretion
O	136	3.42	Posttranslational modification, protein turnover, chaperones
C	212	5.32	Energy production and conversion
G	427	10.72	Carbohydrate transport and metabolism
E	395	9.92	Amino acid transport and metabolism
F	101	2.54	Nucleotide transport and metabolism
H	209	5.25	Coenzyme transport and metabolism
I	131	3.29	Lipid transport and metabolism
P	252	6.33	Inorganic ion transport and metabolism
Q	80	2.01	Secondary metabolites biosynthesis, transport and catabolism
R	342	8.59	General function prediction only
S	204	5.12	Function unknown
-	1192	25.37	Not in COGs

The total is based on the total number of protein coding genes in the genome

### Extended insights

Most phytases of the family *Enterobacteriaceae* family belong to the group of histidine acid phosphatases as judged by their sequence and properties. Three phytase subgroups (AppA-related, Agp-related and PhyK phytases) can be identified within histidine acid phytases based on their substrate specificity and specific activity levels [38]. To gain insight into the phytate-degrading activity of *Pantoea**sp*. 3.5.1 strain, we analyzed its genome for the presence of key genetic factors responsible for phytase activity of *Enterobacteriaceae*.

We detected genes for glucose-1-phosphatase and 3-phytase which are located on the first contig of the assembly (Additional file 3). However, no sequence homology was observed for an *appA*-related gene. Sequence analysis of the 3-phytase gene from *Pantoea**sp*. 3.5.1 revealed maximal homology (77 % nucleotide identity) to *phyK* gene of *P. vagans* C9-1. A high degree of homology of glucose-1-phosphatase gene of 3.5.1 strain was found to glucose-1-phosphatases (*agp*) of *P. vagans* C9-1 (84 % nucleotide identity), *Plautia stali* symbiont (82 %), *P. ananatis* strains and *P. rwandensis* ND04 (81 %), *Pantoea**sp*. At-9b (80 %), and *E. coli* 042 (72 %). Therefore, we show that *Pantoea* sp. 3.5.1 harbors two phytase-encoding genes (*agp*-related and *phyK* phytases) but lacks *appA*-like phytase genes.

**Fig. 3:**
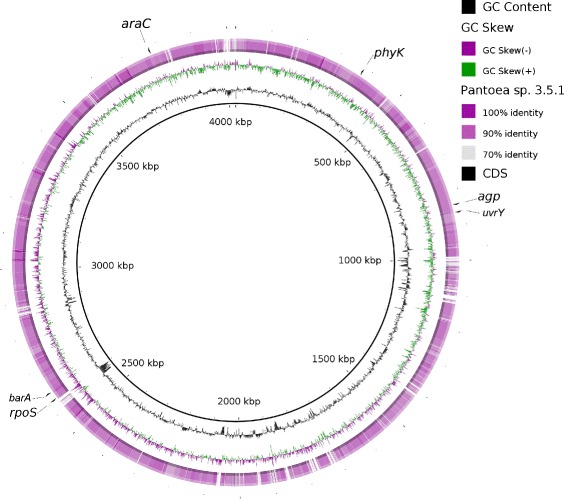
Circular representation of the genome comparison between the *Pantoea sp*. 3.5.1 strain and *P. vagans* C9-1. The inner ring shows GC content (in black) and GC skew (in purple/green). Color intensity within the circle represents the levels of nucleotide homology (from 70 % to 100 %). The external ring shows CDS of two detected phytase genes and the candidate regulatory genes

There is still very little information available in regards to the regulation of phytate-degrading gene expression in bacteria. To date, regulation of two periplasmic phytases of *E. coli* (*agp*-encoded acid glucose-1-phosphatase and *appA*-encoded 3-phytase) have been described in great details [39]. Gene *agp* is constitutively expressed whereas expression of *appA* is induced by phosphate starvation and by transition to stationary phase. Gene *appA* is located within the *appCBA* operon and its regulation occurs by two inducible promoters. We compared phytase genes, their position and context in the *Pantoea**sp*. 3.5.1 genome with *agp* and *appA* genes of *E. coli*. Neither *Pantoea**sp*. 3.5.1 phytase genes (*agp* and *phyK*) have similar locations to genome context of *E. coli* but are comparable with *P. vagans* C9-1 genome context. However we identified two genes which can possibly participate in the regulation of phytase activity similar to the situation in *E. coli*: the *rpoS* gene (RNA polymerase sigma factor RpoS) and *araC*-like gene (DNA-binding domain-containing protein which belongs to the AraC/XylS family). These regulatory genes are active in anaerobic conditions, phosphate starvation and during entry into stationary phase. Thus, the mechanism of phytase activity regulation in *Pantoea**sp*. 3.5.1 might be similar to *E. coli*. Figure 3 shows the results of full genome comparison between the *Pantoea**sp*. 3.5.1 strain and *P. vagans* C9-1 using BLAST Ring Image Generator comparison tool [40]. We also designated the local positions of two detected phytase genes and its possible regulatory genes.

Regulation of intracellular phytase activity has also been investigated in rhizospheric strain of *Serratia plymuthica* IC1270 [41]. It was shown that the GrrS/GrrA system (also known as GacS/GacA and BarA/UvrY) and RpoS factor are implicated in phytase production in *S. plymuthica*. Both genes of GrrS/GrrA two-component signal transduction system were also predicted in genome assembly of *Pantoea**sp*. 3.5.1.

## Conclusions

In the current study, we characterized the genome of the *Pantoea* strain 3.5.1 that was isolated from soils of the Republic of Tatarstan, Russia. The strain exhibits high phytate-degrading activity. Phylogenetically the *Pantoea* strain 3.5.1 is positioned between *P. agglomerans* and *P. vagans*, but the strain 3.5.1 is characterized by phenotypic differences. Thus, it is likely that this strain represents a new *Pantoea* species. In order to improve the understanding of the molecular basis for the ability of *Pantoea**sp*. 3.5.1 strain to hydrolyze phytate we performed detailed genome sequencing and annotation. We also identified three regulatory genes encoding transcriptional factors.

## Additional files

Additional file 1:
**A main spectra profiles (MSP) dendrogram generated by MALDI Biotyper 3.0 software with the 3.5.1 isolate, 26** ***Pantoea*** **reference species and 14** ***E. coli*** **outgroup strains.** Each cluster is indicated by different color. Distance level show the phylogenic distance between the selected genus and species. The strain 3.5.1 is highlighted by box. (JPG 182 kb) Additional file 2:
**Average nucleotide identity (ANI) values calculated between the 3.5.1 genome assembly, 26** ***Pantoea*** **species and 2** ***E. coli*** **strains. Maximum and minimum ANI values are highlighted by yellow color.** ANI values of *E. coli* strains are shown in grey. The corresponding GenBank accession numbers for genome sequences are: NZ_KK403338 (*P. agglomerans* Eh318), NZ_JPOT00000000 (*P. agglomerans* 4), NZ_ASJI00000000 (*P. agglomerans* Tx-10), NZ_JNGC00000000 (*P. agglomerans* 190), NZ_JPKQ00000000 (*P. agglomerans* MP2), NC_017554 (*P. ananatis* PA13), NZ_ASJH00000000 (*P. ananatis* BRT175), NC_013956 (*P. ananatis* LMG-20103), NC_016816 (*P. ananatis* LMG-5342), NC_017531 (*P. ananatis* AJ13355), NZ_JXXL00000000 (*P. anthophila* 11–2), NZ_AVSS00000000 (*P. dispersa* EGD AAK13), NZ_JTJJ00000000 (*P. rodasii* ND03), NZ_CP009454 (*P. rwandensis* ND04), NZ_JFGT00000000 (*Pantoea sp*. IMH), NZ_CP009880 (*Pantoea sp*. PSNIH1), NZ_CP009866 (*Pantoea sp*. PSNIH2), NZ_AJFP00000000 (*Pantoea sp*. Sc1), NC_014837 (*Pantoea sp*. At-9b), NZ_AEDL00000000 (*Pantoea sp*. aB), NZ_JSXF00000000 (*P. stewartii* M073a), NZ_JRWI00000000 (*P. stewartii* M009), NZ_JPKO00000000 (*Pantoea stewartii* subsp. *indologenes* LMG 2632), NZ_AHIE00000000 (*Pantoea stewartii* subsp. *stewartii* DC283), NC_014562.1 (*P. vagans* C9-1), NZ_JPKP00000000 (*P. vagans* MP7), NC_000913 (*E. coli* str. K12 substr. MG1655), NC_002695 (*E. coli* O157:H16 Santai). (DOCX 13 kb) Additional file 3:
**Links and DNA sequences of phytase genes detected in** ***Pantoea sp*** **. 3.5.1 genome.** (DOCX 14 kb)

## Abbreviations

PSM: Phytase screening medium
nr/nt: non-redundant nucleotide
