# Supplementary figures and images for: High-quality draft genome sequence of a new phytase-producing microorganism Pantoea sp. 3.5.1
Source: Stand Genomic Sci. 2015 Nov 11;10:95. doi: 10.1186/s40793-015-0093-y (PMC4642748; doi:10.1186/s40793-015-0093-y)

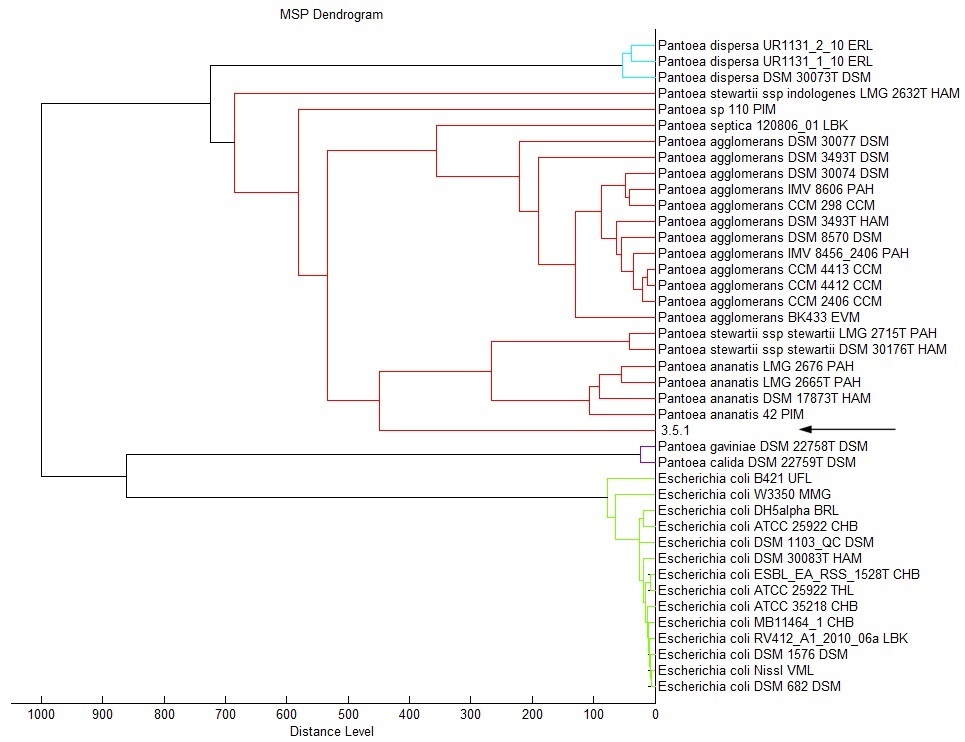

Supplement: Additional file 1: — A main spectra profiles (MSP) dendrogram generated by MALDI Biotyper 3.0 software with the 3.5.1 isolate, 26 Pantoea reference species and 14 E. coli outgroup strains. Each cluster is indicated by different color. Distance level show the phylogenic distance between the selected genus and species. The strain 3.5.1 is highlighted by box. (JPG 182 kb) [file 40793_2015_93_MOESM1_ESM.jpg]
