# Supplementary material for: High-quality draft genome sequence of a new phytase-producing microorganism Pantoea sp. 3.5.1
Source: Stand Genomic Sci. 2015 Nov 11;10:95. doi: 10.1186/s40793-015-0093-y (PMC4642748; doi:10.1186/s40793-015-0093-y)
Supplement: Additional file 2: — Average nucleotide identity (ANI) values calculated between the 3.5.1 genome assembly, 26 Pantoea species and 2 E. coli strains. Maximum and minimum ANI values are highlighted by yellow color. ANI values of E. coli strains are shown in grey. The corresponding GenBank accession numbers for genome sequences are: NZ_KK403338 (P. agglomerans Eh318), NZ_JPOT00000000 (P. agglomerans 4), NZ_ASJI00000000 (P. agglomerans Tx-10), NZ_JNGC00000000 (P. agglomerans 190), NZ_JPKQ00000000 (P. agglomerans MP2), NC_017554 (P. ananatis PA13), NZ_ASJH00000000 (P. ananatis BRT175), NC_013956 (P. ananatis LMG-20103), NC_016816 (P. ananatis LMG-5342), NC_017531 (P. ananatis AJ13355), NZ_JXXL00000000 (P. anthophila 11–2), NZ_AVSS00000000 (P. dispersa EGD AAK13), NZ_JTJJ00000000 (P. rodasii ND03), NZ_CP009454 (P. rwandensis ND04), NZ_JFGT00000000 (Pantoea sp. IMH), NZ_CP009880 (Pantoea sp. PSNIH1), NZ_CP009866 (Pantoea sp. PSNIH2), NZ_AJFP00000000 (Pantoea sp. Sc1), NC_014837 (Pantoea sp. At-9b), NZ_AEDL00000000 (Pantoea sp. aB), NZ_JSXF00000000 (P. stewartii M073a), NZ_JRWI00000000 (P. stewartii M009), NZ_JPKO00000000 (Pantoea stewartii subsp. indologenes LMG 2632), NZ_AHIE00000000 (Pantoea stewartii subsp. stewartii DC283), NC_014562.1 (P. vagans C9-1), NZ_JPKP00000000 (P. vagans MP7), NC_000913 (E. coli str. K12 substr. MG1655), NC_002695 (E. coli O157:H16 Santai). (DOCX 13 kb) [file 40793_2015_93_MOESM2_ESM.docx]

**ANI values calculated between genome of the 3.5.1 strain, *Pantoea* species and *E. coli* strains.**

| **Species** | **ANIm** | **ANIb** |
| --- | --- | --- |
| *Pantoea agglomerans* Eh318 | 86.86 | 85.06 |
| *Pantoea agglomerans* 4 | 86.73 | 85.01 |
| *Pantoea agglomerans* Tx-10 | 86.76 | 85.04 |
| *Pantoea agglomerans* 190 | 86.78 | 85.04 |
| *Pantoea agglomerans* MP2 | 86.84 | 85.11 |
| *Pantoea ananatis* PA13 | 84.08 | 79.41 |
| *Pantoea ananatis* BRT175 | 84.14 | 79.08 |
| *Pantoea ananatis* LMG-20103 | 84.03 | 79.13 |
| *Pantoea ananatis* LMG-5342 | 84.07 | 79.35 |
| *Pantoea ananatis* AJ13355 | 84.21 | 79.29 |
| *Pantoea anthophila* 11-2 | 86.42 | 84.12 |
| *Pantoea dispersa* EGD-AAK13 | 84.35 | 79.18 |
| *Pantoea rodasii* ND03 | 83.82 | 79.39 |
| *Pantoea rwandensis* ND04 | 83.79 | 78.16 |
| *Pantoea sp*. IMH | 84.18 | 74.88 |
| *Pantoea sp*. PSNIH1 | 83.96 | 78.18 |
| *Pantoea sp*. PSNIH2 | 83.72 | 76.54 |
| *Pantoea sp*. Sc1 | 86.19 | 84.22 |
| *Pantoea sp*. At-9b | 83.99 | 78.66 |
| *Pantoea sp*. aB | 86.34 | 84.27 |
| *Pantoea stewartii* M073a | 84.02 | 78.89 |
| *Pantoea stewartii* M009 | 84.02 | 78.38 |
| *Pantoea stewartii* subsp. *indologenes* LMG 2632 | 84.02 | 78.96 |
| *Pantoea stewartii* subsp. *stewartii* DC283 | 84.28 | 79.07 |
| *Pantoea vagans* C9-1 | 86.56 | 84.91 |
| *Pantoea vagans* MP7 | 86.48 | 84.57 |
| *Escherichia coli* str. K12 substr. MG1655 | 83.94 | 72.79 |
| *Escherichia coli* O157:H16 Santai | 83.76 | 72.72 |
