# Supplementary material for: High-quality draft genome sequence of a new phytase-producing microorganism Pantoea sp. 3.5.1
Source: Stand Genomic Sci. 2015 Nov 11;10:95. doi: 10.1186/s40793-015-0093-y (PMC4642748; doi:10.1186/s40793-015-0093-y)
Supplement: Additional file 3: — Links and DNA sequences of phytase genes detected in Pantoea sp . 3.5.1 genome. (DOCX 14 kb) [file 40793_2015_93_MOESM3_ESM.docx]

*agp* (glucose-1-phosphatase) gene in *Pantoea* *sp*. 3.5.1 (by sanger)

<http://www.ncbi.nlm.nih.gov/nuccore/663089996>

*agp* gene in *Pantoea sp*. 3.5.1 (whole genome sequencing) (1 contig)

http://www.ncbi.nlm.nih.gov/nuccore/808897611?from=1643217&to=1644962&sat=19&sat_key=40718289&report=fasta

>gi|808897611:1643217-1644962 Pantoea sp. 3.5.1 contig_1, whole genome shotgun sequence

TTACTGCGCTGCCGTTGCCACTGATTTCGCGTCGTTGCTTTGCGCGGCGGTTGGGGCCTGGCTGGCTGTC

GCGTTGTCGGCTTTAGCTTTTTCAGCCGCGGCTTTCTCAGCGTCTGCTTTCTCTGCTTTGGCATTCTCGG

CCGCTGCTTTATCGGCTGCTGCTTTGTCTGCTTTGGCTTTTTCGGCTGCCGCTTTGTCAGCCGCTGCCTT

ATCTGCTTTGGCTTTTTCAGCGGCTGCTTTGTCCGCTTCAGCCTTCTCTGCTTTGGCTTTATCGGCGTCA

GCTTTCTCTGCCTTCGCTTTTTCAGCGGCTGCTTTGTCCGCTTCAGCCTTCTCTGCTTTGGCTTTATCGG

CGTCAGCTTTGTCGGCCTTCGCGTTGTCAGCCGGCTTATCGGTTGCGCTGTTAGCCGCTGCAGCAGCCGG

TGCCGCATCGCTGTCAGGCTGAGCCGCTGCCGGTTTTGCTGCGGCCGCAGGTTGCATTGGGGTCCCCTGC

AGCGCGCTATTTAACGCCTGCGAGAACTGATCCCAGCTGCAATAGCCATTCGCATCTGTCTGACAACCGG

TCAGTTGCAGAGTAACGCGCTTCGGCGGATTTTTCAGGCTCAGTACATCGGCATCACGCAACTGATCGGC

GGTCTGGTAAACGTACTCGACCTTCAGCAGATCTTTATCATTTTTGGCATCGTGCCAGCGCTCAAATACC

ACCTGACCGCCAATCGGCGTTTTTTCATAGGTATCAGGCAGCTCATACGGCTTCACCTGCAACGCACTCA

GTAAGGAGGCGATGTTGGAGTCGTGGCCAACCATCAGGGTCACTTTTGGCGCATTGGCCTTGTCCTGATC

GACTAACTGGCTACGGATGTAATCCACCAGCGGGGCCGCCACTTCCCGGGCAATCGTCGGGCTGGTGAAC

AGCGTATCCTGATAACCATTTTTAATCGCAGACAGCTCTTTCCACTGCTCAGGCGTTTTGATCTGGCCCC

AGGCAACCTGATCCAGCGGGAAACCTTCATAATATTGCAGCGTAAAGGCATCTATCAGCGAGTTACCCAC

TTTTAACGGGCCACTGACGTTTGGTTCTTTGCCATTTTCCGCGCTGAAGCTGTTCTGGCCGCTGCTCAGA

TCACACTGCTTTTTGTTGTTACAGGCCGGAGAGGATTTGTAGTCAACGATCTTTTCCAGTCGCTGGAAAG

CGGGCTTCAGCGCCAGCTTTCCATTAGCGGCAGCCATGTCAGCCAGCGCTTGCTTGTTGAACGCTTCGCT

GCCATCGGTGATGACCGGATTGAAGATAGGATCCATGGTTCCCATCGCGTCCTGATGGGTGACGGCAACA

TCACAACCCGGGAAGGCACCATTCACAAAGAACTGGGCGGTGGCGACGGTACGCTGCAGGCTATTGGCGT

AAACGAAGACGTTATTGCTATCAGGGCAGCTGCCATTCTGCACCAGACCCTGTTGCGCCAGCCACTGACG

GGTGTAGTTACCCATGTAGATTTCCAGCACGCCGCCTTTGGTGGTCAGTTGTCCACCGGGCACATCCCAT

TGCGGCCAGCTTTTTTTAGTCGACTGCTCCAGCACGCTGCCATTATCCGCCAGCGGCGCACGCAAATTAT

GTCGACTTAACATCAGCACCTGTTGCAGTTGCATATCGCCATCAGCGGCGAAGGCTACCGTTCCGACAGG

TAATGCTGCCAGCACTGATAATGCGCAAAGACTCAGTTTCTTGATCATTGTGCCTATTCCATTCAT

*phyK* (3-phytase) gene in *Pantoea sp*. 3.5.1 (whole genome sequencing) (1 contig)

http://www.ncbi.nlm.nih.gov/nuccore/808897611?from=2166661&to=2167926&sat=19&sat_key=40718289&report=fasta

>gi|808897611:2166661-2167926 Pantoea sp. 3.5.1 contig_1, whole genome shotgun sequence

ATGCGCGCTACCCGATGGTGCCAATGCACGTTAGCCCTGAGTGCACTCTGGCTACTGAGCCTGCCGATGC

AGGCGGCCCCGCTGCCCCATTATCAGTTGGAAAAAGTGGTCGAACTGAGCCGGCACGGCGTGCGGCCACC

GACGCCCGGCAACCGTAAAGAGATCGAAGCCGCCAGCCAGCAGCCGTGGACGGTATGGACCACGGCGGAT

GGCGAGCTGACCGGTCACGGCTACAGCGCAGTGGTCAACAAAGGACGCTGGCAGGGCGAACATTACCGCC

AGCTTGGTTTACTGCCTGCTGGCTGCCCGACAGCAGACGACGTCTATCTGCGCGCCAGCCCGCTCCAGCG

CACCCGAGCCACCGCCCAGGCGATAGCCGACGGTGCGTTTCCGGGCTGTGGCGTACCGATCCATCAGGTG

ACCGGTGAGGTTGATCCACTGTTTCAGAGCGATAAGCTGGCGTTTGCACAAACCGATCCGCAGCAAGAAC

TTGCGGCTAAGCAGCAGAAAGCGGGCGATCTGGCGCAGTTACAGCAGCAACTGCAACCGGCTATTCAGCA

GCTTAAAGCCGCGGTTTGCCCGCCCACGGCGAAATGTGATTTCTTTGATCGCCCATGGCAATTCAAACAG

ACCCGCAGCGGCCACACCTACGTCGAAGGACTGAGCGTGATGGCCAGTATGGTTGAGACCCTGCGGCTGG

GTTACAGCGAAAATCTGCCCGTCGATCAGCTGGCGTGGGGGCATATCACCACCGCGGCGCAAATTACCCG

CCTGCTGCCGTTGCTGACCGCTAATTACGATTTGAGTAATGATCTGCTCTATCAGGCACAGCGGCGGGGA

TCGATTTTACTGCAGGCGATGCTGACCGAAATCGCCAGCAATGCCTCCACTGCCCGCTGGCTGATCCTGG

TGGCGCATGACACCAATATTGCGATGGTGCGTACCCTGATGGGGTTTGACTGGCAGCTGCCGGGCTACGC

GCGCGGCAATATTCCGCCCGGCGGCAGTCTGGTGCTGGAGCGCTGGCGTGACAGCCAGAGCGGCGAGCGT

TTTCTGCGGCTCTACTTCCAGGCACAGAGCCTTGATGGCCTGCGTCAGTTACAGCCGATTGAAGACGCAC

AGGGTTTATTGCGTCAGGAGTGGCATCAGCCAGACTGCAGAATGACCGACGTCGGGCTGCTGTGTCCTTA

TCAATCGACCCTGACCGGCTGGCAACAACGGCTGGACAAGCGCGCCACGCAGCCGGTTTCCATCCGGCTG

CCCTGA
